# Optimal Methods of Documenting Analgesic Efficacy in Neonatal Piglets Undergoing Castration

**DOI:** 10.3390/ani10091450

**Published:** 2020-08-19

**Authors:** Meredith Sheil, Adam Polkinghorne

**Affiliations:** 1 Animal Ethics Pty. Ltd., Yarra Glen, VIC 3775, Australia; 2 Department of Microbiology and Infectious Diseases, NSW Health Pathology, Nepean Hospital, Penrith, NSW 2750, Australia; adam.polkinghorne@health.nsw.gov.au; 3 Faculty of Medicine and Health, Nepean Clinical School, The University of Sydney Medical School, University of Sydney, Penrith, NSW 2750, Australia

**Keywords:** piglet, castration, pain, behaviour, peri-operative, vocalisation, nociception, neonate, anaesthesia, analgesia

## Abstract

**Simple Summary:**

Surgical castration in piglets is widely used in commercial pig production systems; however, it may cause pain and stress to the animal. There is an urgent need to develop effective pain-relieving medications to use for this procedure. Such products must meet high standards of proof confirming that they are effective. This requires undertaking trials to determine the duration and severity of pain that piglets experience during and after castration, and the extent of pain reduction in anaesthetic/analgesic treated piglets. Unfortunately, responses to pain may be transient, subtle, or variably expressed. Furthermore, there is no simple “gold standard” method to measure pain in neonatal piglets. Instead, researchers must rely on using a range of indirect measures of pain of varying reliability. Without understanding the nature of expression of piglet pain, and the reliability of test measures to detect it, there is the potential of misinterpreting trial outcomes. Although there is a high degree of variability in the literature of test methods employed and outcomes obtained, there is nevertheless a growing body of evidence to suggest that some piglet responses to pain induced by castration, are more consistently reproduced and specific to the pain experienced during castration than others. In this narrative review, we examine the potential indicators of pain in neonatal piglets undergoing castration to determine the optimal methods currently available to most accurately detect pain and assess pain mitigation.

**Abstract:**

Analgesic products for piglet castration are critically needed. This requires extensive animal experimentation such as to meet regulatory-required proof of efficacy. At present, there are no validated methods of assessing pain in neonatal piglets. This poses challenges for investigators to optimize trial design and to meet ethical obligations to minimize the number of animals needed. Pain in neonatal piglets may be subtle, transient, and/or variably expressed and, in the absence of validated methods, investigators must rely on using a range of biochemical, physiological and behavioural variables, many of which appear to have very low (or unknown) sensitivity or specificity for documenting pain, or pain-relieving effects. A previous systematic review of this subject was hampered by the high degree of variability in the literature base both in terms of methods used to assess pain and pain mitigation, as well as in outcomes reported. In this setting we provide a narrative review to assist in determining the optimal methods currently available to detect piglet pain during castration and methods to mitigate castration-induced pain. In overview, the optimal outcome variables identified are nociceptive motor and vocal response scores during castration and quantitative sensory-threshold response testing and pain-associated behaviour scores following castration.

## 1. Introduction

A variety of animal husbandry procedures that cause pain to the animal are routinely employed in livestock species as a part of effective animal management systems. A primary example of such a procedure is castration, a technique that involves the removal of the testicles or the removal of testicular function [1]. In pigs, castration is employed in commercial swine facilities for several purposes, including improving meat flavour, preventing unwanted breeding and modifying animal behaviour. It is generally performed in the first week of life in male piglets intended to be kept past sexual maturity. Meat quality is improved by reducing the potential for ‘boar taint’, an unpleasant odour and flavour associated with the presence of androstenone (5α-androst-16-ene-3-one), produced in the testes of intact male pigs following sexual maturity [2]. Castration also reduces the risk of unwanted breeding that can interfere with the maintenance of genetic lines, and assists with management of boars by reducing the presence of aggressive behaviours that pose a welfare risk to other animals and also to the safety of humans interacting with them [3].

Traditional methods involve the use of surgical castration, a rapid (<1 min) method commonly performed by farmers in piglets between 2–7 days of age. The procedure involves restraining the piglet, incising the skin of the scrotum, extracting the testes, and severing the spermatic cords. Antiseptic is commonly sprayed onto the wound, and, less commonly, antibiotics are administered with the piglet finally returned to its sow. The wound is left to heal by secondary intention [1,4,5,6]. As an alternative to this procedure, there is growing interest in raising entire males, and/or the use of immunocastration by an anti-GnRH vaccine, which has shown to be effective in reducing boar taint and increasing growth performance in male pigs [7,8]. Whilst this review focusses on methods of assessing pain mitigation for surgical castration, the reader is referred to comprehensive review articles regarding surgical and non-surgical options and pig welfare [3,6,9]. 

Surgical procedures induce pain via a number of mechanisms [10]. The acute phase is primarily neurally mediated. Tissue incision causes trauma to keratinocytes and nerve fibres at the incision site, resulting in a barrage of nociceptive neural transmission from the damaged tissue to the central nervous system (nociception) inducing spinal reflexes such as the nociceptive withdrawal reflex, and, on reaching the cerebrum, the perception of acute pain and induction of the neuroendocrine response [11]. A second, sub-acute or prolonged inflammatory phase arises, primarily due to local release of various mediators in response to tissue damage, that promote ongoing pain or pain hypersensitivity against thermal, mechanical, and chemical stimuli [12,13]. Pro-nociceptive mediators such as ATP, glutamate, kinins, cytokines, tropic factors, and prostaglandins activate primary afferent neurons directly or indirectly to enhance nociceptive signal transmission to the central nervous system [14,15,16,17]. Prostaglandins derived from the arachidonic acid cascade are implicated in the production of inflammatory pain, and in sensitising nociceptors to the actions of other mediators. Bleeding and coagulation due to tissue injury are closely associated with the initiation of inflammation resulting in reflex erythema and acute pain responses. Kallikrein released during coagulation produces bradykinin, a strong allogenic factor [18]. Degranulation of activated mast cells results in the release of proteases, cytokines, serotonin, and histamine into the extracellular space. These substances sensitize primary afferent neurons to produce hyperalgesia [19]. Sensitization of peripheral and central neuronal structures amplifies and sustains postoperative pain [10,15,19].

Consistent with this, piglet castration is reported to cause pain and stress to the animal involving (i) discomfort and stress prior to the procedure due to handling and restraint; (ii) acute pain and stress during the procedure itself associated with incision of the scrotum, separation of the tissue to release each testicle, followed by severing of the spermatic cord; and (iii) post-operative pain and/or discomfort in the hours and days following the procedure [1,6]. Despite this, historically, castration has been typically performed without any pain relief, including in North America [20] and the EU [5]. In a detailed survey of 26 European countries, undertaken as part of the PIGCAS project (Attitudes, Practices and State-of-the-Art regarding Piglet Castration in Europe) in 2009, in the European Union [5] it was estimated that 79.3% of the about 98 million male pigs were castrated and analgesic use was reported as “very rare” or “never” in most EU member countries surveyed. Over the past decade, however, welfare concerns and ethical objectives have led to a drive to develop effective pain relief strategies for piglet castration, along with strategies to support the phasing out of the procedure where possible. In 2010, for example, the ‘European Declaration on alternatives to surgical castration of pigs’ was agreed, stipulating the intention that from 1 January 2012, surgical castration of pigs should only be performed with prolonged analgesia and/or anaesthesia. From 2018, the declaration stipulates that the surgical castration of pigs should be phased out altogether. This has seen progress with non-surgical alternatives along with exploration of a range of different anesthetic/analgesic options for piglet castration. These include the use of general anaesthesia (with CO_2_, isoflurane, or injectable agents); the use of injectable local anaesthesia (such as lignocaine or procaine) administered by a combination of subcutaneous scrotal and intra-testicular (i.t.), or infundibular injection 5–15 min prior to the procedure; and/or the use of non-steroidal anti-inflammatory (NSAID) medications generally also administered 20 min prior to castration, via intra-muscular (i.m.) injection or oral administration [3,6]. An updated survey of 24 different European countries in 2016 [21] identified significant progress; however, concluded that the deadlines were far from being met. Whilst 6 of the countries had the practice of raising entire males, an average 80% of pigs continued to be surgically castrated in the remainder. The average percentage of piglets receiving immunocastration was 2.7%, 5% of the male pigs surgically castrated received anaesthesia and analgesia while 41% received analgesia alone [21] and 54% received no anaesthesia or analgesia. As analgesia alone ameliorates post-operative but not acute procedural pain, the development of practical and effective anaesthesia for the procedure was identified as an urgent priority.

The challenge faced by stakeholders in this field is to identify options that are effective in mitigating pain but are also safe, practical and economically sustainable for use in commercial swine facilities. Few medications are specifically approved for this use in piglets, and many must be used off-label under veterinary prescription [3]. General and local anaesthesia may be effective to provide pain relief during the procedure, but not after [22,23,24,25], and may require specialized equipment or veterinary administration, precluding practicality or commercial viability in many situations. Although some countries allow farmers to administer injectable local anaesthesia, this is not widespread [21]. Furthermore, injected sedatives or anaesthetics often require time to take effect resulting in negative welfare impacts, such as due to the pain of injection and/or the need for double handling. There may also be negative consequences if agents induce post-operative sedation due to interference with feeding and increased risk of crushing [3,6]. Although data on this is conflicting, NSAIDs may assist to mitigate post-operative inflammatory pain [26], however, they appear to offer little effective alleviation of pain during the procedure or in the early minutes and hours following the procedure [24,25,26,27,28,29] when pain is most acute. As NSAIDs take time to reach therapeutic effect, they commonly require administration well before castration, thus also resulting in negative welfare impacts due to pain of injection and the need for double handling of piglets. Hence, currently there is a critical therapeutic gap in availability of practical farmer-applied methods of delivering safe and effective peri-operative anaesthesia. Our group is investigating the use of a combination topical anaesthetic and antiseptic formulation, which may be farmer-applied during the procedure, (administered via intra-operative wound instillation), as a method to mitigate acute peri-operative pain in piglets. This has proven effective to alleviate castration pain in lambs and calves and is now widely used on farms in Australia [30,31]. Administered immediately following skin incision, the topical anesthetic formulation (containing 5% lidocaine, 0.5% bupivacaine, cetrimide, and 1:2000 adrenalin), can act rapidly, within 30 s, to anaesthetise the wound and the exposed cordal tissues prior to severing the spermatic cords [32], which is considered the most painful part of the procedure. The longer acting local anaesthetic bupivacaine is included in the formulation to assist in providing extended post-operative sensory analgesia [33]. Extensive animal experimentation, such as to confirm safety and efficacy, is required for regulatory approval and authorization for use in piglets. Prior to commencing such studies, we performed a review of methods of assessing analgesic efficacy in neonatal piglets to identify those most valid, sensitive, and specific for the assessment of pain and the efficacy of analgesic medications.

Proof of anaesthetic/analgesic efficacy is challenging in neonatal piglets. There is no one gold standard or validated measure of pain in piglets. Signs of pain in neonatal piglets can be subtle and variably expressed, and readily confounded by extraneous variables, particularly when required to be examined in the field setting (as opposed to in a laboratory) as is a standard requirement for regulatory approvals. Nevertheless, it is generally accepted that piglets react to stimuli in a number of ways including: physiologically, behaviorally, and through resistance movements and vocalization [1,6]. On this basis, a range of outcome variables have been used to assess piglet pain during and following castration, and to assess amelioration of pain due to use of local anaesthetics or analgesia. These include; (a) physiological responses during the procedure [22,25,28,29,34,35,36,37,38,39,40,41,42,43,44,45,46,47,48,49,50,51,52,53,54,55,56,57,58], (b) nociceptive motor responses during the procedure [28,29,32,48,50,55,59,60]; (c) vocal responses during the procedure [22,25,28,29,32,47,50,60,61,62,63,64,65,66,67,68]; (d) mechanical sensory testing in the minutes and hours following the procedure [32,33,45] and; (e) post-operative pain-related behaviours in the minutes and hours following the procedure [22,23,24,27,28,29,35,45,47,51,54,58,63,65,66,67,68,69,70,71,72]. More recently, newer technologies have been explored including (e) facial expression [45,65,71], and (f) infra-red thermography (IRT) [29,40,46,52]. Unfortunately, the methods used to examine analgesic efficacy in the reported literature have varied considerably between investigators, and the detail and quality of reporting has been highly variable, precluding the ability to make standardized assessments of the validity of each measure. As highlighted in previous reviews on this topic [26,73,74], this variation in the methods has impeded efforts to develop science-based guidelines for pain management protocols for castration.

To be valuable as indicators of pain mitigation, measures must be capable of consistently detecting a significant difference in pain-associated responses during and/or following castration as compared with pre-operative values, and/or as compared between castrated and non-castrated piglets. Secondly, variables must optimally be physiologically and/or clinically relevant to the evaluation of the type of pain being measured e.g., intraoperative pain or post-operative pain. Ideally, these measures (i) must be practically measured within the study without being confounded by the assessment of other variables; and; (ii) have the ability to be measured using an analytical method or measurement device/subjective assessment tool that has sufficient validation.

In the current review, we summarise literature on the currently available methods for assessing peri-operative pain in surgically castrated neonatal piglets and provide a critical analysis of the outcome variables identified to ascertain those that most closely meet these criteria. It is anticipated that this critical analysis may assist the future development of more standardized methods and optimise (reduce and refine) future analgesic efficacy trials in this field.

## 2. Physiological Measurements of Pain in Piglets

Physiological responses occur in response to pain and stress, including activation of the hypothalamus-pituitary-adrenal axis (HPA-axis) and sympathetic nervous system (SNS), and release of opiate neuropeptides. This acts to increase the metabolic rate in preparation for flight or fight as well as mediate the inflammatory response and mitigate pain. Adrenalcorticotrophic hormone (ACTH) is released by the pituitary and acts on the adrenal gland. Cortisol and adrenalin are released and, in turn, result in an increase in the level of glucose and lactate in the blood. Activation of the SNS may result in an increased heart rate and blood pressure and reduced skin temperature as blood is diverted to muscles and vital organs. β-endorphins (endogenous opioid-neuropeptides) are released from the anterior pituitary and act on opiate receptors in the peripheral and central nervous system to induce analgesia principally through effects on mu-opioid receptors. Indicators of the HPA axis and SNS activation, or β-endorphin release are thus often used as indirect measures of pain.

These physiological responses; however, are not specific to pain. They may be triggered by stress alone, and/or by tissue trauma (such as induced by surgical incision), even in the absence of pain. Surgical studies reveal that animals under a general anaesthetic increased cortisol and ACTH production, irrespective of the animal’s sensation of pain [75,76]. Haemorrhage alone is known to result in an increase in ACTH, cortisol, β-endorphin concentration, as well as tissue content of pro-inflammatory cytokines; (including tumour necrosis factor-alpha (TNF-a) and interleukin-1alpha (IL-1a), IL-6 and IL10), and opiates have a proposed role in regulating the hemodynamic response to blood loss [77]. In a porcine model of abdominal surgery, for example, a standardized laparotomy without visceral involvement was performed on 24 anaesthetized pigs. Surgery gave rise to dramatic increases in plasma ACTH and cortisol (*p* < 0.01 and *p* < 0.001, respectively) within 15 min of incision, while animals were still under full general anaesthesia [75]. The activation of the HPA axis, and inflammatory cascade in response to surgical tissue trauma is generally termed the surgical stress response [78], and plays an important role in haemostasis and fluid homeostasis, immune defence, endogenous pain mitigation, and wound healing [76].

Similar to other surgical procedures, piglet castration results in an acute physiological response with activation of the HPA-axis and SNS, and opiate neuropeptide release. Prunier et al. [4] reported that castration of piglets induced significant (*p* < 0.05) increases in ACTH from 5 to 60 min, cortisol (from 15 to 90 min), and lactate (from 5 to 30 min) following the procedure, although no significant changes in blood glucose were observed. These authors hypothesised that glucose may not increase in neonatal piglets due to lack of glycogen stores. There is also a very rapid and transient increase in plasma adrenaline, followed by a longer lasting increase in plasma noradrenaline [4] as well as an increase in heart rate, blood pressure, and other signs of activation of the SNS such as reduced skin temperature that have also been reported [4,53,66]. Elevated β-endorphin levels have been reported in piglets castrated via cutting, but not via tearing the spermatic cord, despite equivalent rises in cortisol, as well as motor and vocal responses during the procedure [50]. This was hypothesised to be due to the increased risk of blood loss when cutting as opposed to tearing the cordal tissues.

Highlighting concerns over interpreting such physiological markers as being indicative of pain rather than in response to surgical tissue trauma, comparisons of anaesthetised and non-anaesthetised castrated piglets have found no significant difference in stress hormone responses [48,49]. Plasma cortisol, ACTH, and β-endorphins did not differ significantly between the anaesthetised and non-anesthetised castration groups indicating that tissue trauma (with inflammatory mediator release) and/or blood loss, rather than pain, is primarily responsible for the physiological HPA-activation and opiate neuropeptide response. Cortisol was reported as “not a sensitive tool to judge castration stress” in piglets castrated under general anaesthesia [49]. This indicates that variability in wound size, blood loss, and a piglet’s neuroendocrine and immune response to wounding may all have a greater impact on cortisol levels than pain in piglets undergoing castration.

Furthermore, activation of the HPA axis and SNS may occur simply through handling and restraining piglets. Marchant-Forde et al. [50] reported that cortisol and β-endorphin levels were increased 45 min following the procedure in castrated piglets versus sham-handled controls (*p* < 0.1), however this was associated with a significant difference in the duration of handling and restraint, and was no longer evident when these factors were taken into account. Hay et al. [51] did not find differences in urinary levels of corticosteroids and catecholamines over the 4 days following surgical castration of piglets, as compared with sham-handled controls. This was considered most likely due to the short-lived activity of the adrenal and sympathetic axes [4]. Lonardi et al. [52] reported a short-lived increase in cortisol levels in castrated versus sham-handled animals at 20 min but not at 3–24 h following the procedure. Lactate and glucose levels were not significantly different between the two groups. Sutherland et al. [22] reported increased cortisol levels in castrated versus sham-handled piglets 30–120 min, but not 180 min or 24 h following procedure, however the study involved prolonged handling of piglets for blood collection and/or administration of anaesthetic treatments prior to castration, and the actual duration of restraint and handling was not documented for each piglet to allow group comparisons. Substance P (SP), however, was not significantly different between groups. SP is a neurotransmitter released directly from damaged nerve fibres at the site of tissue damage and is associated with increased pain perception and, hence, used as a biomarker of pain [79]. Other studies have reported that castrated piglets tended to have higher cortisol levels than sham-handled pigs, however this did not reach statistical significance at the *p* < 0.05 level [47,50]. Interestingly, where duration of restraint was controlled to be equivalent between groups, there were also no significant differences between castrated and sham-handled piglets in plasma levels of pro-inflammatory cytokines; TNF-ɑ and interleukin-1beta (IL-1β), or on acute phase proteins C-reactive protein (CRP), serum amyloid A (SAA) and haptoglobin (Hp) and Moya et al. [54] concluded that pro-inflammatory cytokines and acute phase proteins did not provide relevant information on the physiological consequences of castration in neonatal piglets. Together, these data suggest that handling alone may induce a physiological response similar to that of castration in neonatal piglets. Despite the significant impact that the duration of restraint and handling may have on results, this variable is not always detailed in study reports or included as a variable in analyses.

Local anaesthetics and NSAIDS act to block pain via different mechanisms. This has important implications regarding interpreting the validity of biomarkers of HPA axis, neuroendocrine and/or inflammatory cascade activation as indicators of pain in this setting. NSAIDs mitigate pain via blockade of the conversion of arachidonic acid to prostaglandins by cyclooxygenase enzymes (COX), preventing activation of the inflammatory cascade and release of pain-inducing inflammatory mediators. Prostaglandins also directly stimulate ACTH and cortisol release. Separate from mitigating pain, NSAIDs thus also may directly mitigate the humoral aspect of the surgical stress response to tissue trauma [80,81]. A reduction in cortisol following NSAID administration, may be anticipated to indicate a collateral reduction in production of prostaglandins and other associated pain-inducing inflammatory mediators in piglets post castration, and hence also an associated decrease in pain. Hence, cortisol or ACTH levels may provide an indirect biomarker of pain in piglets following NSAID administration. This is not the case for local or general anaesthetics, however.

Local anaesthetics act by blocking nerve fibre conduction of pain signals. These prevent pain sensation via local or central nervous system effects, without primary effect on the humoral/inflammatory response to tissue trauma or associated HPA-axis activation. Biomarkers associated with the surgical stress response may thus be elevated, even although pain induced by them is blocked. Such variables are thus unlikely to be reliable indicators of pain in animals administered local or general anaesthesia. An additional confounding factor in the case of local anaesthetics is that, in many cases, these are administered in combination with adrenalin. This is to enhance local anaesthetic effects and minimize risks of systemic absorption. Adrenalin and nor-adrenalin, may have centrally and/or peripheral effects to stimulate corticotrophin releasing hormone and increase the breakdown of proopiomelanocortins into ACTH and β-endorphins [82,83,84]. Exogenously administered adrenalin may thus confound markers of endogenous HPA-axis and SNS activation and opiate-peptide production in castrated piglets.

In view of these factors, it is not surprising that studies investigating the impact of local anaesthesia or analgesia on physiological parameters in piglet castration have shown highly variable and, at times, apparently conflicting results (Table 1). The more consistent results are seen with the use of NSAIDs. Compared with piglets castrated without analgesic treatment, significantly reduced plasma cortisol and/or ACTH levels have been documented in NSAID-treated piglets at 30 min [35,38,41,45], 60 min [35,37,45,46], or up to 4 h post-procedure [35,37]. Others however, have reported no significant (*p* < 0.05) effect of NSAIDs administered prior to [25,36,39] or at the time of the procedure [22,42], on cortisol and/or ACTH, nor acute phase reactants, Hp, SAA, and/or CRP. Bates et al. [40] reported significantly greater amount of prostaglandin E_2_ (PGE_2_) inhibition at 10h, and from 30–100 h post castration in piglets which had nursed from meloxicam- as opposed to placebo treated sows prior to procedures. Cortisol and SP concentrations, however, were not significantly different (*p* < 0.05) between the two groups. O’Connor [74] and associates concluded a weak recommendation for use of NSAIDS for pain alleviation in piglets 1–24 h post-castration following a systematic review of available trial data, based principally on impact on cortisol. In the same review, NSAIDs were not found to have any impact on vocalisation to suggest an effect to mitigate procedural pain, which is discussed further below. Together, these data support the conclusion that some NSAIDs may have activity to reduce the inflammatory response and HPA-axis activation resulting from tissue trauma in piglets in the hours following castration, consistent with their known mechanism of action. Where cortisol and ACTH levels are reduced post castration (despite equivalent handling duration between treatment and control groups), this may be indicative of the efficacy of NSAIDs to mitigate post-operative inflammatory pain.

By contrast, as expected, the majority of studies have found little or no impact of either local or general anaesthesia on markers of the tissue trauma/inflammatory response to piglet castration and resulting activation of the HPA axis. Pre-emptive use of local anaesthesia via intra-testicular (i.t.) or infundibular injection, or via topical wound instillation, has been associated with reduced cortisol levels as compared with untreated animals in some trials [25,40,45], while not in others [25,47,55,56], or only where local anaesthetics and NSAIDs have been used in combination [20]. As detailed above, the lack of efficacy of local or general anaesthesia to reduce cortisol or ACTH does not, however, represent lack of efficacy to mitigate pain. These agents act via a different mechanism and mitigate pain via blockade of neural transmission. Neural markers of pain mitigation, such as the expression of the c-fos gene and its protein product, Fos, in neurons of the spinal cord [85], are significantly reduced when piglets are castrated under effective local or general anaesthesia, as compared with piglets castrated without anaesthesia [43,44]. Furthermore, this is associated with a dramatic reduction in the nociceptive physiological [4,53], motor and vocal response to castration [22,25,29,32,48,55,59,60,66,67]. Additionally, reduced post-operative hyperalgesia has been documented in local anaesthetic-treated piglets [32,33]. Together, these factors are considered to indicate that biomarkers of activation of the HPA axis, and inflammatory response lack specificity for pain mitigating effects of local and general anaesthetics, and are poor indicators of pain in piglets castrated under general or local anaesthesia [1]. They are similarly not suited to comparative efficacy trials with NSAIDs.

Based on this review, it is concluded that biomarkers of activation of the HPA axis, SNS, opiate neuropeptides and immune response, lack specificity as indicators of pain associated with neonatal piglet castration, and are confounded by the physiological response to restraint and to tissue trauma. They may provide some indication regarding the efficacy of NSAIDs to reduce post-operative inflammatory pain; however, are very poor markers of potential pain mitigating effects of local or general anaesthetics.

## 3. Nociceptive Motor Responses during Piglet Castration

Piglet castration without anaesthesia induces protracted violent struggling and escape behaviour in piglets during the procedure [48]. This piglet motor response is usually accompanied by a loud vocal response and is attributable to the nociceptive withdrawal response to acute pain induced during the procedure. It is referred to in the literature by a variety of terms including ‘escape attempts’ [50]; ‘defense behaviour’ [55,60] or; ‘resistance movements’ [29,59]. Measurement of the nociceptive motor response is typically conducted by use of a variety of methodologies [86] including (i) ordinal scales [60] (ii) focal assessments [28,55], (iii) a visual analogue scale (VAS) [29], or; (iv) the use of a numerical rating scale (NRS) [32,48]. Regardless of the methods used, analysis of the nociceptive motor responses of piglets consistently detects a marked and significant increase in castrated versus sham-handled animals, and successful mitigation of this response through use of general or local anaesthesia, indicative of sensitivity to detect pain mitigating effects (Table 2).

Numerous studies have demonstrated that the piglet nociceptive motor response to castration is significantly increased in piglets undergoing castration as compared with sham-handled controls and/or following the application of effective local or general anaesthesia (Table 2). Marchant–Forde et al. [50] reported that castration triggered significant escape attempts in piglets undergoing castration compared to sham-handled controls. Focal sampling observations revealed that the piglet’s nociceptive motor response often involved a sequence of sequential leg kicks in an attempt to escape, followed by a pause. Injectable anaesthesia (i.e., 2% Lignocaine) applied via intra-testicular or infundibular injection with an effective wait time has been shown to reduce the relative proportion of resistance movements from the entire period of fixation, including during the cutting of the spermatic cords, which elicits the greatest response and is considered to be the most painful step of the procedure [59]. A subsequent study investigating lignocaine effectiveness also confirmed less resistance movements during castration in piglets pre-injected with 10 mg/mL lignocaine into each testicle as compared to untreated animals. By contrast, pre-emptive i.m. administration of an NSAID did not result in a significant reduction in nociceptive motor response [28].

To investigate the efficacy of topical anaesthesia to mitigate piglet castration pain when instilled into the wound and allowed a 30 s wait time, our group recently employed a method in which piglet castration was recorded on videotape, and the nociceptive motor response was graded off-line by a blinded trained observer using an NRS (0–2, based on nil, partial, or vigorous full body response) including scoring at four specific time points during the surgical procedure (i.e., during traction of each testicle and severance of each spermatic cord). Piglets were settled at the time of commencing procedures. Nociceptive motor response scores were increased at all four time points in untreated piglets, and were also shown to be significantly reduced in animals treated with topical anaesthetic via wound instillation with 30 s dwell time [32]. Together, this literature is considered to indicate that assessment of nociceptive motor withdrawal response can provide a consistent, sensitive and repeatable method for documenting piglet pain responses during the castration procedure, and the efficacy of pain management strategies.

## 4. Vocal Responses during Piglet Castration

A review of the literature indicates that some changes in piglet vocalisation (i) can be detected during surgical castration, (ii) can be moderated with the use of anaesthesia and; (iii) are considered to be indicative of pain (Table 3). Although piglets commonly vocalise when they are handled, and particularly when restrained, the literature indicates that during castration piglets may squeal more often, more loudly and/or at a higher frequency than piglets that undergo sham-handling [1,25,29,47,50,60,61,62,63,64,65,68]. Castration is reported to produce changes in piglet vocalisation sound parameters that are comprehensively different to those detected from handling alone [67]. A wide range of parameters have been employed to measure piglet vocal response including measurement of; duration, energy or loudness (dB), peak frequency or pitch (Hz), or highest energy (Hz), vocalisation rate, and/or the percent of piglets that vocalised. Parameters that describe a single event in a call, such as peak level or peak frequency are considered to provide more consistent results than parameters that describe an average, such as weighted frequency and main frequency [67]. Most recently, specifically designed software (STREMODO; Stress Monitor and Documentation System, Forschungsinstitut für die Biologie Landwirtschaftlicher Nutztiere, Dummerstorf, Germany) [64,87] has been developed to detect stress vocalisations in piglets. This uses linear prediction analysis [88] to differentiate stress calls, non-stress calls, or background noise.

Studies have reported that piglets during castration produced more high-frequency calls (>1000 Hz), (referred to as screams [67]), than non-castrated controls. Pulling and severing of the spermatic cords lead to the greatest vocalisation response, greater than those normally emitted during handling and restraint as well as during the initial incision [67,68]. Vocalisation responses were also used to compare the castration procedure itself with cutting or tearing of the spermatic cord found to have little difference on the duration of responses [50]. Interestingly, intra-muscular injection of analgesics induces vocalisations of similar power (dB), frequency (Hz), and energy as that induced by pulling and tearing the spermatic cords during castration, and of significantly greater power (dB), frequency, (Hz) and energy than skin incision [65].

The majority of studies identify that local and general anaesthesia are effective in mitigating piglet vocal response to castration. Piglets castrated without local anaesthesia produce a higher number of screams with higher frequencies compared to piglets castrated with anaesthesia [29,60,66,67]. Hansson et al. [29] used a decibel meter during castration to record the highest vocal intensity level (dB) of piglets castrated with and without a local anaesthetic (lignocaine). Piglets castrated without the local anaesthetic produced calls of a significantly higher intensity than those administered lignocaine. Leidig et al. [60] summed the total duration of stress calls relative to the total time of the procedure, finding that duration of vocalisations of piglets receiving intra-testicular anaesthesia with injectable procaine was half of that emitted by piglets without anaesthesia. Animals that have received local anaesthetic injection to the testicle on one side vocalise less when the anesthetised testicle is removed than the non-anaesthetised testicle, although there was wide variability from animal to animal [89]. Trials examining the impact of NSAID administration at or prior to castration, however, have uniformly reported little to no impact on piglet vocal responses during castration [22,25,26,36,74] compared to piglets castrated without NSAID treatment.

Despite the overall consistency of reported outcomes, the actual metrics reported by authors are very diverse and reporting of measures of variation is poor, such that it is difficult to combine these data or quantify the effect of anaesthetic interventions on vocalisation [26,74]. A confounder to studies that rely on the quality of vocalisation responses to assess pain in piglets is that, in most cases, these findings have been recorded in rooms acoustically isolated from farrowing pens where piglet castration usually takes place. Since regulatory safety and efficacy trials require demonstration in the field situation, the sensitivity of pig vocalisation measurements and the consistency of results needs to be considered against the normal background noise levels, and confounding factors of a farrowing pen in a commercial farm setting. The presence of the sow and littermates can have confounding effects on piglet vocal responses. In view of these factors, it may be anticipated that analysis of vocal responses may not be as sensitive an indicator of pain in regulatory field trial settings as in acoustically separated research environments.

We recently developed a modified method for quantifying piglet vocal responses in the on-farm setting [32]. Piglet vocal response was recorded using a decibel meter as well as time-stamped videotape recording. Off-line analysis by a blinded technician allowed generation of standardised decibel/time waveform recordings for each piglet, on which the time of various specific procedural events were able to be marked. This allowed comparison of the peak (dB) and total auditory response (area under the dB/time waveform curve (AUC)) of each piglet, during specific procedural event-time periods (e.g., piglet vocal response during traction and severing of each cord). This provided consistency and specificity to the measurement period. Using this technique, we identified that both the peak dB and AUC recording were significantly reduced in piglets (n = 20) treated with topical anaesthesia instilled to the wound followed by a 30 s wait time, as compared with untreated piglets (n = 20) during traction and severing of the first cord. A trend effect was evident for traction and severing the second cord however statistical power was affected by increased variability. This finding was in contrast to a previous report [47] in which vocal responses in castrated piglets treated with topical anaesthetics or an NSAID were compared with untreated controls (n = 10 per group) using the STREMODO system. No measurable difference had been recorded between treatment and non-treated castrated groups in this trial. This may have been due to lack of sufficient dwell time allowed for efficacy of the topical anaesthetic agents employed, and/or insufficient power. More recently, we commissioned a further trial examining vocal response to castration following wound instillation of a topical anaesthetic formulation (with 30 s dwell time) (n = 44 per group) using peak dB and area under the dB/time waveform (as above) to compare vocal response to castration between treated and untreated piglets. With increased power, a significant reduction in vocal response (peak dB and AUC) to traction and severing of both the first and second spermatic cords was recorded. (Sheil, M; unpublished observations, manuscript in preparation).

In summary, it is considered that with careful application to ensure targeting of the measurement period to coincide with the time points of pain generation, and avoidance of confounding factors (particularly duration of restraint or recordings), measures of piglet vocalisation in response to castration including; the peak dB, total vocal response (such as area under the dB/time waveform), the frequency (Hz) of call with the highest intensity (dB (A)), rate of high frequency calls (>1000 Hz) or stress vocalisations using the STREMODO system, appear to provide a relatively consistent and sensitive method of assessing procedural pain associated with castration, and pain mitigation in neonatal piglets.

## 5. Post-Operative Pain-Related Behaviours

In general, measures of behaviour have proven to be more reliable indicators of pain than physiological measures in animals following castration [1,51]. In other animal species, behaviours such as decreased or abnormal locomotion, turning the head towards the rump, abnormal postures including prostration (standing or sitting with head below the shoulders), “hunching” (standing with kyphosis), “stiffness” (lying with legs tense and extended or walking with a stiff gait), increased or reduced movements of the tail are considered indicators of pain resulting from castration [30,31,90,91,92]. More diffuse and variable responses may occur in neonatal animals, however, due to immaturity of neuronal pathways involved with pain processing [93].

Behavioural disturbances have also been examined in neonatal piglets following castration. A review of the literature however reveals that in piglets, these behavioural changes may be subtle, transient and/or variably expressed, such that findings are not always reproducible. In some cases, contradictory results have also been reported (Table 4 and Table 5). Behavioural assessments usually involve either direct quiet observation and scoring of piglet behaviours by trained blinded observers, or continuous time-lapse video-recording with off-line scoring either using event monitoring software or trained blinded observers. Assessments typically include observations of piglet; (i) posture (lying, standing, sitting, etc.), (ii) location (under heat, in contact with the sow or pen mates versus in isolation), and (iii) activities, including “non-specific” behaviours (sucking, sleeping, walking, playing, exploratory or aggressive behaviour, etc., which may be divided into “active” and “inactive” behaviours) and “pain-specific” behaviours. This latter category, first detailed by Hay et al. [51] based on pain-specific behaviours reported in other species, includes; “prostration” (standing or sitting with head down below shoulder height), “huddled up” (ventral lying with at least three legs tucked up), “tremors or trembling”, “spasms” (localised muscle spasm), “stiffness” (lying with legs tense and extended), “tail wagging” and “scratching” (rubbing the rump along the floor or walls, also called “scooting”). Authors have additionally included standing in “hunched” posture (i.e., with kyphosis) or walking with a stiff or abnormal gait [23,45,52]. Observations may be made by “scan sampling” (i.e., recording the general posture, position, and behavioural activity of the piglet, with frequent repetition (e.g., every 1–10 min), over a predetermined time period (generally 2–3 h in the morning and afternoon of each assessment day), and/or by “focal assessment” (scoring the presence or absence of “pain-specific” behaviours at a number of predetermined time points). As incidences of individual pain-specific behaviours are low, aggregation of “pain-specific” behaviours is commonly employed to derive a “total” or “global” pain score for each piglet over specific time periods [28,45,51,54].

Using these methods, abnormalities of behaviour have been documented in the early minutes and hours after piglet castration, principally consisting of a low magnitude increase in “pain-specific” behaviours and/or isolation. Although the majority of these behaviours are short-lived (i.e., observed with the greatest frequency in the first 30 min to 1 h following castration), some particular behaviours such as increased tail wagging and/or scratching tend to develop later in the post-operative period and have been observed to be increased for up to 2–5 days post-procedure in some studies [51,65,67], although not in others [23,25]. Overall in review, when comparing castrated piglets with sham-handled controls, variation in general postures and non-specific behaviours have been marginal and/or conflicting, and are generally not considered reliable indicators of piglet pain [27,51,94].

Early studies identified a number of behaviours thought to be indicative of pain in piglets, including changes in posture, position, and nursing behaviour, with reduced standing and increased lying away from heat, and reduced nursing in the early hours (3–6 h) following the procedure as compared with uncastrated controls, effects that were ameliorated by use of lignocaine local anaesthesia prior to castration [70,72]. A subsequent study [63], however, reported differently, documenting decreased lying, increased sitting, and increased nursing in piglets post-castration as compared with uncastrated controls. In all cases, however, the authors reported that effects, although statistically significant, were marginal and/or of low magnitude. Hay et al. [51] introduced a detailed ethogram for behavioural assessment of piglets post-castration. This included recording a range of indices of piglet posture and position, as well as ‘non-specific’ behaviours (such as suckling, walking, running, sleeping, playing, exploring, aggression), “pain-specific” behaviours (detailed above) as well as “social cohesion” (isolation and desynchronization). Using this ethogram and scan sampling over 5 days, in a study of piglets 5 days of age (n = 84) following castration, increased “pain-specific” behaviours were documented involving greater incidences of prostration, stiffness, trembling, huddled-up posture and tail wagging as well as increased social isolation and de-synchronisation, during the first 2.5 h following castration in castrated versus sham-handled piglets. Scratching and tail wagging were increased at later time points and remained elevated for 2–4 days. There were no significant changes in other variables, and it was concluded that general postures changes and non-specific activities were not reliable indicators of pain in piglets post-castration [51]. A number of studies have used similar ethograms and/or assessment of “pain-specific” behaviours to investigate post-operative piglet pain since this time (Table 4 and Table 5). These have reported changes in “pain-specific” behaviours and social isolation, generally detectable only during the earliest assessment periods up to 180 min following castration. A recent study examining shorter time intervals identified significant changes in “pain-specific” behaviours were only present over the first 30 min post-castration [45]. Most studies have reported minimal [51,70] or no significant effect on suckling, and all studies have reported no effects of castration on piglet weight gain when performed on neonatal piglets > 3 days of age (Table 4). Longer-term behavioural effects have been variably reported. Hay et al. [51] reported scratching was increased with maximum frequency from 24–48 h post-operatively, and tail wagging was increased for 4 days. Wemelsfelder and van Putten [61] also documented increased tail wagging in the days following castration in 4-week old piglets. However, piglets in both these trials had also undergone prior tail docking, and it was hypothesised that prolonged tail wagging could be related to exacerbation of tail stump hyperalgesia. Viscardi et al. [65,69] recorded a significant increase in tail wagging, peaking at 24 h in non-tail-docked piglets, with no significant difference in scratching behaviour. Others have reported no significant differences in scratching or tail wagging in castrated piglets as compared with non-castrated controls up to four days post-castration [25,54].

Pre-treatment with local anaesthetic or NSAID analgesic has been shown to result in significant differences in certain pain-related behaviour in treated piglets less than 2 weeks of age in some trials [45,47,72], but not others [24,27,69]. McGlone et al. [72] reported that although the changes in behaviour were only minor, piglets castrated without local anaesthetic were observed to display significantly reduced standing, increased lying, and reduced nursing behaviours compared to piglets administered lignocaine via injection prior to castration. Hansson et al. [29] documented reductions in total “pain-specific” behaviours in piglets administered both lignocaine and meloxicam (but not alone) prior to castration as compared with untreated piglets. Sutherland et al. [47] examined the behavioural responses of piglets after castration and found that untreated animals spent significantly more time lying without contact (isolation) compared with piglets given topical anaesthetic via wound instillation during the procedure. In contrast, an alternative study [27] reported that lignocaine injection prior to castration resulted in increased “pain-specific” behaviour in the first hours after castration as compared with sham or unhandled controls, or NSAID-treated piglets. This was predominantly due to a significant increase in tail-wagging and huddling up in the early hours after the procedure, with increased tail-wagging remaining evident over the first 3 days. It was hypothesised that either the effect of the lignocaine wore off so quickly that it had no post-operative analgesic effects or the sensation of the lignocaine wearing off may have resulted in increased tail-wagging in piglets. Yun et al. [23] also reported increased tail-wagging in the first 10 min post castration piglets in piglets castrated under lignocaine or general anaesthesia. In this case, however, tail-wagging was also similarly increased in non-castrated piglets but not in piglets castrated without anaesthesia or analgesia, as compared with pre-operative values. Increased tail wagging in the early hours following the procedure was also reported following post-operative use of lignocaine hydrochloride spray to the wound [24] as compared with untreated castrated piglets. It was hypothesised that the high proportion of alcohol in the product and/or its acidic pH may have contributed to afferent nerve sensitisation. Such effects if present, may be preventable using buffering agents [95,96] and/or formulations that do not include high alcohol concentrations. Increased tail wagging was not evident in the early hours following castrated in piglets treated via wound instillation with a combination topical wound anaesthetic and antiseptic formulation (containing lignocaine along with bupivacaine), as compared with untreated piglets or pre-operative values [47] (M. Sheil, unpublished observations, manuscript in preparation). In this situation, it could be hypothesised that bupivacaine provides longer-acting sensory nerve blockade that may mitigate any sensation as the shorter-acting lignocaine wears off. Using focal assessment and an amalgamated global “pain-specific” behaviour score, Keita et al. [28] documented reduced scores at 2 and 4 h post-castration between Meloxicam-treated piglets versus those without treatment, however, there were no significant effects at 30 min, 1 h, or 24 h. Little or no difference in pain-related behaviour was seen after castration performed with or without general anaesthesia [23,47]. This is not unexpected, as general anaesthetics act primarily to prevent pain perception at the cortical level, however, they have little impact on the local cytokine response to tissue trauma that induces afferent nerve sensitisation and the development of post-operative pain, as detailed above. Hence, post-surgical inflammatory pain develops as general anaesthetic effects wear off.

It is notable that the majority of studies that have identified changes in “pain-specific” behaviours in the early hours following castration have been performed using direct observation with scan sampling and/focal assessment as opposed to continuous video recording techniques. From a scientific perspective, continuous behavioural observation is generally considered the gold standard for pain evaluation in animals, as it allows detection of deviation in normal behaviour and is considered to have the sensitivity to detect subtle or short duration behaviours [97]. Performed using video recording and off-line analysis, it also avoids the potential for confounding by observer effects on animal behaviour, and other limitations of live observations, such as reduced number of duration and frequency behaviours observed. However, video-recording may be impaired by 2-dimensionality, parallax error, and shadowing. Furthermore, behaviours may be missed when animals are grouped, hidden, or off-screen, such as may occur frequently in a farrowing pen. Such factors may all contribute to reduce sensitivity of video-recording methods to the detection of subtle behavioural changes such as are seen in neonatal piglets in the early post-operative period. It is notable that no significant differences in “pain-specific” behaviours between castrated and sham-handled neonatal piglets were evident in the first 2 h following castration in trials using video-recording techniques [65,69] as opposed to those using direct observation [29,45,51,54]. Data from these trials suggest that video-recording techniques may have high sensitivity to detect tail-wagging, however, lower sensitivity to detect other “pain-specific” behaviours such as tremors, spasms, huddling up, prostration or stiffness in neonatal piglets. Although two trials [22,47] using direct observation methods also failed to detect significant differences in “pain-specific” behaviour in piglets post-castration as compared with sham-handled piglets these trials only examined a narrow range of “pain-specific” behaviours (scooting and huddling up) as compared with the full range detailed by Hay et al. [51] and involved relatively low piglet numbers per group. This suggests that the studies may have been underpowered, and/or that important pain-specific behaviours such as tremors/trembling, prostration, spasms, stiffness and tail-wagging may have been missed. There are limited validation studies on behavioural methodologies to detect piglet pain associated with castration, however, Hay et al. [51] compared 10 min scan samples to continuous sampling on pain behaviours associated with castration and reported no difference in results when utilizing a scan or continuous methodology. Additionally, Burkemper [24] has reported low inter-observer error following observer training for direct observation of pain-associated behaviours. New studies are underway [98], using video recording techniques with event monitoring software, and comparing continuous versus scan sampling at various intervals, to better understand the sensitivity and repeatability of this method. New or alternative methods of behavioural assessment such as examining gait, locomotor performance, and latency to move are also being explored [23,52].

On this basis, our group recently examined pre- and post-operative pain-related behaviour in castrated piglets 3–5 days of age with and without wound instillation of topical anaesthesia during the procedure, across two separate trial sites (M. Sheil, unpublished observations). Direct observation using trained blinded observers was used, with scan assessments of posture and position (including pain-specific postures and positions, such as prostration, huddled-up, hunched standing, stiffness and isolation) as well as behaviours (including “non-specific” and “pain-specific” behaviours) which were recorded every 10 min for 3 h in the morning and 2 h in the afternoon; pre-castration and over the first 36 h post-castration. In addition, focal assessments of “pain-specific” behaviours were separately made pre-castration and at 1, 15, 30, 60, 90 min and, 2, 4, 6, 8, 24, and 30 h post-castration. Our results accord with those of Gottardo et al. [45], who, using similar methods, reported increased total “pain-specific” behaviour evident predominantly in the first 30 min after castration, which was mitigated by pre-administration of analgesic medication or post-surgical topical anaesthetic medication. Additionally, using similar methods, Hansson et al. [29] reported reduced total “pain-specific” behaviours in the first 70 min period following castration in neonatal piglets administered both NSAID and local anaesthetic prior to castration. These results suggest that this method currently provides the most consistent, repeatable method of identifying acute post-operative pain, and documenting pain-mitigation in the early minutes and hours following castration in neonatal piglets. We did not find a difference in “pain-specific” behaviours between groups at later times, based on focal sampling, however, scores at later times were similar to pre-operative values. This is consistent with findings reported by Yun [23] and associates, who, reported increased pain-related behaviours in the first 10 min and to a lesser extent at 60–70 min following castration, but not at other time points measured over 24 h in castrated piglets as compared with non-castrated piglets and/or pre-operative values. Using scan and/or focal assessment methods, Keita et al. [28], Hansson et al. [29] and Burkemper et al. [24]. have previously reported relatively increased “pain-specific” behaviour at later time periods following castration in untreated as compared with analgesia/anaesthesia-treated piglets, however pre-operative baseline values were not reported in the piglets under study, nor were sham-handled groups included.

Interestingly, we observed that most piglets were sleeping (~55%) or suckling (~20%) during baseline (pre-operative) scan observations. A prominent increase in piglet sleeping was evident the afternoon following castration. A similar finding has been reported by Viscardi et al. [65,69] who similarly compared piglet behaviour pre- and post-castration. An increase in piglet sleeping has otherwise been infrequently reported as a post-operative behavioural disturbance in piglets although it is, however, a well-documented response to aversive stimulation in neonates [99,100] and neuroactive steroids such as allopregnanolone, and endogenous neuropeptides such as β-endorphin, released in response to stress, are known to have potent sedative properties [101,102,103,104,105]. The majority of previous trials have examined piglet behaviour comparing castrated with sham-handled animals, rather than using a piglet’s pre-castration behaviour as its own control. As handling and restraint are aversive to piglets (resulting in a neuro-endocrine and opiate-neuropeptide stress response), increased sleeping following handling and restraint may be common to both castrated and sham-handled animals. This could explain a lack of difference in sleep between sham-handled and treatment groups in previous trials. Kluivers-Poodt et al. [27], for example, reported a large proportion (70–75%) of piglets sleeping during scan assessments the afternoon following castration or sham-handling, however, there were not significant differences between castrated and sham-handled piglets. Trends for increased lying, with reduced standing, walking, exploring etc., and/or reduced active behaviours following castration, where reported, (Table 4 and Table 5) could all be consequent upon an increase in piglets sleeping following handling, rather than being indicative of post-castration pain. It is interesting to note that buprenorphine administration prior to handling or castration resulted in a significant reduction in inactive behaviours (including sleep) and increased active behaviours in the 8 h following castration or sham-handling in neonatal piglets [67]. Buprenorphine is reported to disrupt sleep and decrease adenosine concentrations in sleep-regulating brain regions of the Sprague Dawley rat, [101] such that it could be hypothesised to have similarly disrupted sleep following aversive stimulation in piglets. A sedative response to aversive stimulation in piglets, if present, could explain the relatively low proportion of piglets exhibiting “pain-specific” behaviours over the same period, and contribute to the challenges detecting pain (and determining the efficacy of pain mitigation strategies) using behavioural observation methods at these later time points. Increased tail-wagging and scratching are the most consistently reported behavioural disturbances evident during later time periods, particularly in docked piglets, however, scratching may not be seen to a significant extent for 24 h.

It is concluded that the expression of pain in neonatal piglets is subtle and confounded by behavioural responses to handling stress. Pain assessment is confounded by the lack of a validated assessment method, which has resulted in variability in the methodological approach taken in trials to date, and in the reported results. This is concerning because of the potential to underestimate both the degree of pain experienced by neonatal piglets and the ameliorating effects of analgesic medicines. In review, direct observation of piglet behaviour, pre- and post-castration using frequent scan and/or focal assessment and an ethogram that includes and is targeted to the observation of known “pain-specific” postures, positions and behaviours, including; tremors/trembling, spasms, prostration, huddled up or hunched posture, stiffness, tail-wagging, scratching, and isolation currently appears to provide the optimal method to most consistently identify a difference in acute pain-induced behaviour between castrated and non-castrated piglets, and investigate the potential efficacy of analgesics or anaesthetic medicines in the acute post-operative period. Tail wagging and scratching are the most consistently reported behavioural anomalies at later time points and appear to be equally well documented via continuous recording with off-line analysis or direct observational methods. These variables may however indicate irritation or itch rather than pain, particularly if present in the absence of other pain indicators (such as hyperalgesia) and appear to be exacerbated in piglets that are tail-docked.

## 6. Mechanical Sensory Response Testing

Quantitative sensory testing is a long-established and validated method of assessing the efficacy of local anaesthesia and wound analgesia in laboratory research and clinical settings [108]. The flexion reflex, or nociceptive withdrawal reflex, is a reflex response to a nociceptive stimulus resulting in the withdrawal of a limb or body part from a painful stimulus, which may be abolished by effective local anaesthesia or analgesia. In the setting of tissue injury, the release of chemical mediators such as SP, prostaglandins, and bradykinin involved in the inflammatory response, increase sensitisation of neurons to nociceptive signals resulting in the development of hyperalgesia and a reduction in the threshold for the nociceptive reflex response [109]. Afferent nerve sensitisation resulting in hyperalgesia is considered the primary pathological mechanism underlying the development of post-operative inflammatory pain [10]. The threshold for eliciting the flexion reflex may be clearly measured, including in rats [110], and pigs [111] and used to assess the development of hyperalgesia and the efficacy of anaesthetic or analgesic interventions. The reflex is evoked by stimulation of small calibre A6 or C fibre primary afferents which transmit noxious information. The absence of the reflex response and/or a measurable change in the reflex threshold may be detected using a variety of stimuli including needlestick, heat pads, calibrated or electronic Von Frey Filaments and/or pressure algometry.

Von Frey filaments or ‘hairs’ are a set of calibrated filaments that bend when a certain pressure is reached, allowing a reproducible mechanical stimulus to be delivered, graduating from that inducing a light-touch sensation through to a pain-weighted stimulation of skin or tissues. Electronic von Frey anaesthesiometers are also available. Using an electronic von Frey anaesthesiometer, Herskin and Rasmussen [112] have described thresholds of mechanical nociception in the pelvic limb of pigs, using four categories of behavioural response (from slight leg movements to kicking) to detect and grade the threshold response. In addition to laboratory studies in humans, pigs, and experimental animals, modified techniques have been developed for use “in the field” for assessment of pain and pain-alleviation in association with surgical husbandry wounds in livestock species. Applied to skin in proximity to a wound at time points before and after surgery, an animals response to a fixed light touch and pain-weighted Von Frey filament stimuli can be graded (via NRS) from a nil response (0) through to; a local twitch (1), or partial (2) or full body (3) nociceptive withdrawal response. The development of hyperalgesia lowers the threshold for a response, resulting in a greater response score to application of the same stimulus. This method has provided a sensitive, consistent and repeatable method of documenting the development of post-operative wound hyperalgesia and assessing the efficacy of topical or local anaesthetic-induced wound anaesthesia/analgesia in a range of livestock species following surgical husbandry procedures, including mulesing, tail docking and/or castration in lambs [31,113,114], castration and dehorning in calves [30,114]. Using this technique, a heightened nociceptive motor response to stimulation of a surgical husbandry wound has been documented in the minutes and hours following the procedure, in lambs, calves, and piglets, as compared with sham-handled animals, and/or with pre-operative assessments, indicative of the development of post-operative hyperalgesia. Pre-operative use of injected local anaesthetic (lignocaine) and/or immediate post-operative use of topical local anaesthetic (Tri-Solfen^®^) applied to the wound has resulted in a significant reduction in nociceptive withdrawal responses evident within 1–3 min of application, and continuing in the minutes and hours following the procedure, indicative of significant wound anaesthesia or hypoaesthesia [31,32,33,113,114,115,116]. Where present, this has been associated with evidence of reduced post-operative pain-related behaviour in treated animals over the same period.

In pigs, this method has been shown to elicit similar and measurable responses to those reported in human studies, and is sensitive to the effects of local anaesthetic agents [111] (Table 6). Von Frey filaments have been employed in studies to assess the efficacy of pain mitigation in piglets following surgical castration [32,33]. Wound sensitivity testing involved the use of von Frey monofilaments of weights 4 g and 300 g and an 18-gauge needle to stimulate the wound and surrounding skin at predetermined sites prior to treatment and then at defined periods of time afterward. Involuntary nociceptive motor responses were scored using an NRS as above. Topical anaesthesia using a lignocaine, bupivacaine adrenalin combination formulation was found to provide rapid wound anaesthesia and subsequent effective wound analgesia, with treated pigs displaying significantly reduced responses compared to untreated animals [32,33] within one minute and continuing 2–4 h post operatively, and showing similar responses to wound stimulation as sham-treated piglets [33]. Pre-operative lidocaine injection (scrotal and intra-testicular), also induced early wound hypoaesthesia, with reduced responses as compared with untreated piglets for up to 1 h following castration.

As an alternative to von Frey filaments and needlestick stimulation, pressure algometry involves applying a force to a point and measuring the pressure at which a withdrawal response is elicited using a pressure algometer. Both A and C fibers mediate pain induced by pressure stimulation [108]. Acute pain in piglets following castration and the impact of local and topical anaesthesia (tetracaine) has also been assessed by pressure algometry [45]. Efficacy of pain relief was assessed prior to and during a 300 min period after castration by scrotal skin pressure sensitivity, amongst other methods. Increasing pressure was applied to a designated point on the skin of the scrotum adjacent to the incision site and the pressure point by which a physical or vocalisation response was elicited was recorded. Results were consistent with behavioural results in which reduced pain-related behaviours documented in the first 30 min following the procedure were more prominent in NSAID than topical tetracaine-treated piglets. While one study investigating wound sensitivity in calves found a good agreement between both Von Frey filament stimulation and pressure algometry [30], other comparative studies in piglets (M. Sheil, unpublished observations) found pressure algometers were relatively insensitive due to the soft nature of the scrotal tissues. The pressure device induced discernible indents or trauma to the soft tissues at the site without consistently eliciting a response. Janczak et al. [117] examined factors affecting mechanical (nociceptive) thresholds in piglets and the stability and repeatability of measures of mechanical (nociceptive) thresholds in piglets when using a hand-held algometer to examine potentially confounding factors. These investigators reported that mechanical (nociceptive) thresholds can be used both for testing the efficacy of anaesthetics and analgesics, and for assessing hyperalgesia in chronic pain states in research and clinical settings; however, identified that in piglets age and weight affected responses to pressure algometry, particularly in the first week of life.

Whilst the number of reports of quantitative nociceptive response testing in neonatal piglets post castration are limited, direct sensory testing using needlestick and von Frey stimulation with NRS grading of the nociceptive withdrawal reflex response, has thus to date proven consistent, repeatable, sensitive and specific to the pathophysiological process generating pain, and is concluded to provide the optimal method currently available for assessing post-operative hyperalgesia secondary to peripheral afferent nerve sensitisation following castration in neonatal piglets.

Quantitative sensory testing allows assessment of an animal’s response to noxious stimuli, (nociception) as an indicator of the peripheral afferent nerve sensitisation that underlies the development of post-operative pain but does not necessarily indicate the more complex cortical perception of pain, i.e., the experience of pain in the absence of a direct stimulus. Combining the use of QST with the assessment of spontaneous pain-related behaviour is recommended when assessing pain mitigation strategies, such as to provide evidence of reduced experience of pain, as well as reduction in its primary underlying pathophysiological mechanism.

## 7. Other Measures of Pain

Several alternative methods to assess perioperative pain in piglets have also been described.

A piglet grimace scale (PGS) was recently proposed as an alternative method to assess castration and tail docking pain in piglets [71]. Similar methodologies have previously been developed and validated for a variety of livestock species, including sheep [118] and horses [119]. The piglet PGS was developed following analysis and comparison between still images of piglet faces captured at various stages after surgical castration and the concurrent presence/absence of behaviours indicative of piglet pain. Facial actions indicative of pain were considered to be (i) drawing back of the ears from a forward position; (ii) the presence of a bulge of skin on the snout in response to cheek tightening; and (iii) orbital tightening [71]. This initial study reported a strong correlation between PGS score and behavioural activity in animals in the first several hours after castration [71]. Some doubts about the robustness of this method to consistently detect pain in neonatal piglets currently exist though. In a follow-up study applying the PGS, there were not significant differences between sham-handled and castrated piglets, and a potential cofounder in the form of piglet body weight was identified, suggesting that facial grimacing may also indicate weakness or stress related to lower body weight rather than pain [65]. It was also documented that administration of buprenorphine significantly reduced facial grimace scores as compared with both sham-handled and untreated castrated piglets. As buprenorphine also reduced sleep and increased the activity state of both sham-handled and castrated piglets, this suggests the possibility that piglet activity state (as opposed to pain) may also impact facial grimace scores. The second issue relates to inter-user operability with one study [45] revealing that the PGS method was too unreliable for use in comparative evaluation of piglet pain. It failed to show consistent inter-observer reliability in scoring in 2 of the measures while the 3rd measure, orbital tightening, did not differentiate the positive and negative control. This is therefore considered to be a promising new development however further experience and validation are needed for use in in-field trials of piglet castration pain and analgesic efficacy.

Infra-red thermography (IRT) measurement of skin temperature has also been used as a non-invasive method to assess pain responses in piglets with conflicting results reported [29,40,42,46,52]. Animals in pain lose heat from the body’s periphery, measurable by IRT, due to activation of the SNS causing vasoconstriction and redirection of blood flow to the internal organs [120]. Thus, piglets experiencing significant pain via surgical castration should display quantifiably lower skin temperatures than sham-castrated piglets or piglets treated with effective pain mitigation strategies. Consistent with this hypothesis, skin temperature dropped to a greater extent immediately following castration in untreated piglets as compared with sham-handled animals and those administered both lidocaine and meloxicam prior to castration [42]. In addition,, cranial temperatures in piglets castrated and tail-docked following nursing from meloxicam-treated sows were found to be significantly higher than temperatures recorded in piglets which had nursed from placebo-treated sows up to 60 h after castration [40]. However, there were no significant differences between groups in IRT values at other sites (ear or snout-tip). Furthermore, these results conflict with an earlier study that found ear temperatures were increased in untreated piglets compared to piglets treated with meloxicam (i.m.) and/or intra-testicular lidocaine prior to castration [29]. Skin temperature measured using IRT at the wound site did not differ significantly between groups. Similarly, a separate report examining the effect of NSAID treatment administered to the sow prior to husbandry procedures in piglets, found decreased skin temperatures in piglets of sows treated with NSAID compared with piglets from placebo-treated sows at 2 and 4 h post-procedure, with no difference between groups at 1 h, or from 7–24 h following the procedure [46]. These results conflicted with eye temperature recordings in the same cohort which were increased at 1 h in the NSAID versus the placebo group, but not significantly different between groups from 2 and 4 h or up to 30 h following procedures. These investigators also identified significant temperature differences between male and female piglets and a seasonal variation in skin and eye temperature recordings.

A confounder to IRT measurements in this setting is that body temperature is also affected by the post-surgical inflammatory response (i.e., not only the SNS response to pain). Lonardi et al. [52], examined rectal temperature and eye temperature in castrated versus sham-handled piglets and documented that there was an increase in both rectal and eye temperature over time following castration or sham-handling and, although some values were numerically higher in castrated animals, there were no significant differences between the two groups. The increase in eye temperature correlated with the increase in rectal temperature. It was noted that body temperature is reported to increase in response to anxiogenic or stress-inducing stimuli or injury (surgery and trauma) secondary to endogenous inflammatory activation [121,122,123]. Inflammatory mediators such as TNF-α and IL-1β are considered the main endogenous pyrogens [121]. These endogenous pyrogens are increased in piglets 3 h after castration or sham-handling [54]. It was considered that this may explain the tardive hyperthermia observed in the study in both castrated and handled piglets, although other external factors interfering with body temperature, such as exposure to heat lamps or time from milk intake could not be excluded.

NSAIDs have anti-inflammatory and associated direct anti-pyretic effects and thus may have a lowering effect on temperature that may confound the assessment of any effect due to mitigation of the SNS response to pain. This is further complicated by differences in doses and methods of administration employed in trials, as well as pharmacokinetic parameters of different NSAIDs [124] and a relative lack of detail regarding effective therapeutic range for anti-inflammatory effects in neonatal piglets. General anaesthetics may also have direct effects on body temperature and peripheral vasodilation. Local anaesthetics generally do not have significant direct anti-pyretic effects, however, are commonly administered with adrenalin, which may cause peripheral vasoconstriction and similarly confound skin temperature assessment. Yet another confounder is the relationship between the body’s temperature and circadian rhythms with day/night cycles influencing body temperature results in meloxicam-treated and untreated castrated piglets [40].

In view of the lack of consistency in results to date, and multiple confounders, thermography does not currently appear to provide a reliable indicator of pain in neonatal piglets’ post-castration, particularly following administration of local anaesthesia with adrenalin. Thermography may be more reliable for assessment of pain or pain mitigation in non-surgical settings.

## 8. Conclusions

Sensitive, specific, and well-validated methods of assessing pain provide the cornerstone for developing effective analgesic medications. Unfortunately, there are few such methods available for assessing pain associated with castration in neonatal piglets. This is confounded by the neonatal piglet’s physiological response to restraint, handling, and surgical stress due to tissue trauma, and the seemingly subtle, and short-lived expression of pain in the post-operative period. An understanding of the strengths and weaknesses of currently available methods for pain assessment is critical to identifying and developing effective pain mitigation strategies in neonatal piglets. Employing methodologies that lack specificity or reliability risks underestimating both piglet pain, and the efficacy of pain-relieving medications, and creates welfare concerns associated with unproductive or counter-productive research. In the absence of a validated “gold standard” method of assessment, use of a number of different methods are required and, indeed, this is a foundational requirement for any treatment method seeking regulatory approval. This review has discussed the potential strengths and weaknesses of a range of currently available methods of pain assessment in the context of examining the efficacy of different anaesthetic and/or analgesic treatment options in field trial settings.

Based on the detailed review of different methods for assessing perioperative pain associated with surgical castration of piglets, this review concludes that:

- there is a relatively short-lived (0–3 h) physiological response to castration in neonatal piglets; however, physiological parameters lack specificity for pain, and may be significantly confounded by the surgical stress response as well as response to restraint and handling. They do not provide a reliable method for assessment of pain-alleviating efficacy of general or local anaesthetic interventions. Due to differences in mechanisms of action, these parameters may however provide a more reliable method to assess efficacy of NSAIDs where confounding variables are adequately controlled.
- pain control during piglet castration may be evidenced most consistently and reliably by a reduction in spontaneous nociceptive motor response during the procedure such as by NRS or VAS scoring of intensity of motor response.
- measurement of piglet vocal response to castration provides a second method for assessing pain control in piglets during the procedure. Variables including; peak dB, total vocal (dB/time) response, the frequency (Hz) of call with the highest intensity (dB (A)), and the rate of high-frequency calls (>1000 Hz), or stress calls as documented by STREMODO, appear to provide the most consistent or reliable parameters for detection of a significant reduction in vocal response.
- for both nociceptive motor and vocal response assessments care should be taken to ensure piglets are settled prior to commencing procedures and recordings to provide a consistent baseline. It is also suggested that measures be adopted to minimise confounding factors (such as piglet responses to restraint and/or extraneous environmental stimulation) by targeting/limiting the assessment period as closely as possible to the time of acute pain generation. This is considered particularly important if studies are required in the field situation as opposed to acoustically separated environments.
- post-operative pain control is most effectively evidenced by documenting a combination of reduced peripheral afferent nerve sensitisation with an associated reduction in pain-related behaviour.
- peripheral nerve sensitization (hyperalgesia) is currently most reliably and consistently documented in neonatal piglets using nociceptive threshold testing with Von Frey and needlestick as opposed to pressure algometry.
- post-operative pain-related behaviour may be variable, subtle, and short-lived. Careful planning of variables and time points to be measured as well as power is required. The most consistently reported behavioural changes indicative of acute pain in piglets post castration include; “huddling up”, “prostration”, “hunching”, “stiffness” (lying or of gait), “spasms”, “tremors/trembling”, “isolation”, “tail-wagging” and “scratching”(as defined above), which are most evident in the first 30 min to 1 h following castration. The most consistently reported abnormalities of “pain-specific” behaviour at later timer points are tail-wagging and “scratching”. It is noted however that both tail-wagging and scratching may indicate itch or irritation as opposed to pain, particularly if present in the absence of other indicators of pain (such as presence of hyperalgesia) at these later time points. They may be exacerbated in piglets that are also tail docked.
- other methods in development such as facial grimace scores and thermography, hold promise in many situations however do not currently appear to provide a reliable or consistent method of documenting pain or pain mitigation in neonatal piglets following castration.

It is hoped that this review may assist the future development of more standardized methods of assessing pain mitigation in neonatal piglets, assist investigators to optimise (reduce and refine) future analgesic efficacy trials in this field, and support the development and evaluation of innovative effective and practical approaches to improve piglet welfare where surgical castration is still utilised in commercial pig facilities worldwide.

## Figures and Tables

**Table 1:** Summary of studies investigating physiological (neuro-endocrine and inflammatory) responses during piglet castration.

Authors	Piglets N, Age	Castration Experimental Groups	Significant Findings
Prunier et al. [57]	18, 7–9 days	Castrated without analgesia/anesthesia (CAST), sham-handled (SHAM) or no handling	↑ ACTH; (5 to 60 min), cortisol (15 to 90 min), and lactate (5 to 30 min) in CAST animals. No effect on glucose.
Marchant-Forde et al. [50]	328, 2–3 days	CAST (cut or tear), SHAM	Blood sampling immediately before and at 45 min, 4 h, 48 h, 1- and 2-weeks post procedure. 45 min post-castration—↑ cortisol (trend) in CAST vs. SHAM piglets. And ↑β-endorphin (trend) in cut vs. tear and SHAM piglets. Significantly longer duration of procedure noted in CAST piglets vs. SHAM piglets, however.
Moya et al. [54]	40, 5 days	CAST, SHAM (controlled for time of restraint)	Blood sampling before (0 h) and 1, 2, 3, and 4 h after procedures (cortisol, TNF-a, and IL-1b) and before (0 h) and 12, 24, 48, and 72 h after procedures (CRP, SAA, and Hp). ↑cortisol trend only (*p* < 0.1) in CAST vs. SHAM and no statistically significant difference between groups (NSD) for TNF-a, IL-1b, CRP, SAA, or Hp.
Lonardi et al. [52]	32, 4 days	CAST, SHAM	Blood sampling 1 h before and at 20 min, 3, 5, and 24 h after procedures. ↑ cortisol in CAST vs. SHAM animals 20 min but not 3–24 h post-castration; ↓ lactate and glucose (SHAM and CAST) 3–24 h post-castration.
Carroll et al. [58]	90, 3, 6, 9, and 12 days	CAST, SHAM	Blood sampling before and at 0.5, 1, 1.5, 2, 24, and 48 h after castration. ↑cortisol for 0.5–2 h after procedure CAST > SHAM and ↑cortisol in older versus 3-day-old piglets.
Hay et al. [51]	84; 5 days	CAST; SHAM; (Animals previously tail-docked)	NSD between CAST vs. SHAM animals during 4 days of urinary measurements
Keita et al. [28]	90; mean 5 days	CAST; SHAM; NSAID (NSAID = Meloxicam (M) i.m. 10–30 min prior to castration).	30 min post castration—↑ cortisol in CAST and M versus SHAM. ↓ cortisol and ACTH in M vs. CAST group, (ACTH in M group similar to SHAM). NSD for Hp at 24 h.
Langhoff et al. [35]	245; 4–6 days	CAST, SHAM, NSAID (NSAID = M, flunixin (F), metamizole (MET), carprofen (C)), or saline i.m. 15–30 min prior)	Blood sampling before and at 30 min, 1, 4 and 24 h following procedures. ↑ cortisol in CAST piglets 1 and 4 h post castration; ↓ cortisol in all NSAID vs. CAST piglets; (↓ cortisol in M and F vs. CAST at 30 min, 1 h and 4 h; NSD vs. SHAM treated animals at 1 h).
Reiner et al. [36]	N/A	SHAM, NSAID (M or F)	↑ cortisol in NSAID vs. SHAM piglets 30 min post-castration
Zöls et al. [37]	78; 4–6 days	CAST, SHAM, NSAID (M) i.m. prior	↑ cortisol in CAST vs. NSAID and SHAM piglets 1, 4 (but not 28) h post castration.
Schwab et al. [38]	130; <7 days	CAST, SHAM, NSAID (Ketoprofen, (K) i.m. 10–30 min prior)	30 min post-castration—↑cortisol and ACTH CAST > NSAID > SHAM piglets.
Wavreille et al. [39]	66; 5–6 days	CAST, SHAM, NSAID (Tolfenamic acid (T) or M)	NSD CAST vs. SHAM or M; ↑cortisol 30 min post-castration in T-pigs.
Bates et al. [40]	10 sows; 60 piglets; 5 days	CAST(M)-(piglets from M-treated sows), CAST(*p*)-(piglets from placebo-treated sows)	↑ PGE_2_ inhibition, 10 h and 30–100 h post (castration and tail docking and iron injection) in CAST-M vs. CAST-*p* piglets. NSD between groups for plasma cortisol and SP. (Peak cortisol occurred 1 h post procedures).
Marsálek et al. [34]	36, 4 days	CAST, SHAM, local anaesthesia (LA) (LA = Lignocaine(L) with Noradrenalin (N-adr), administered i.t. 3 min prior)	↑ cortisol CAST and LA vs. SHAM at 1 h after castration. (L with N-adr did not modify cortisol concentrations).
Saller et al. [55]	54; 3–7 days	CAST, ±NaCl, L2%, Procaine (P) 4%, Bupivacaine (B) 0.5%, Mepivacaine 2% 20 min prior; SHAM (all with low flow isoflurane)	CAST: ↑ Heart rate + blood pressure. NSE on cortisol, Adr, Nor-Adr or chromogranin A. (LA did not modify cortisol or catecholamine concentrations, despite significant reduction in heart rate, blood pressure and nociceptive motor responses)
Zöls et al. [59]	124; 4–6 days	CAST, SHAM, LA (LA = *p* i.t. 15 min prior)	↑ cortisol in CAST and LA vs. SHAM piglets 1, 4 (but not 28) h post-castration. (P did not modify cortisol concentrations)
Courboulay et al. [41]	96	CAST, SHAM, NSAID (K), LA (L).	↑ cortisol at 30 min in L and CAST vs. K and SHAM.
Kluivers-Poodt et al. [25]	160, 3–5 days	CAST, SHAM, NSAID (M), LA(L), L and M (L-i.t.and s.c. M-i.m. administered 15mins prior)	Cortisol, lactate glucose and creatinine kinase (CK) measured before and 20 min following procedures. ↑ cortisol all grp vs. SHAM. ↓ cortisol L vs. CAST and M. NSD any treatment groups, for lactate, glucose or CK.
Hansson et al. [29]	564; 1–7 days	CAST; NSAID (M); LA (L with adr), LA and NSAID (Administration L with adr -i.t. 3–30 min prior, M-i.m. post castration).	Trend to reduced SAA in NSAID-treated piglets.
Bonastre et al. [42]	120; 4–7 days	CAST, SHAM, SHAM and NSAID (M), CAST with M, CAST with LA(L), CAST with L and M, CAST with L and B, CAST with L, B and M (Administration; L and B i.t. 20 min prior; M i.m. immediately post-castration).	↑ cortisol (20 min) in all groups except SHAM and CAST with L+M; ↑ glucose (20 min) in all groups except SHAM and CAST and L.
Nyborg et al. [43]	NA	CAST, LA. (LA= L and B administered intrafunicularly (bilateral) and subcutaneously prior to castration)	↑ cFos protein (spinal cord) in CAST vs. LA piglets
Svendsen [44]	20	CAST, CAST and LA, CAST and CO_2_/O_2_ general anaesthesia (GA)	↑ cFos protein (spinal cord) in CAST vs. LA and GA piglets
Gottardo et al. [45]	196; 4 days	CAST; SHAM; NSAID (M, K or T); CAST with topical anaesthesia (TA) (TA = 2% or 6% topical tetracaine hydrochloride prior and applied to wound immediately post-procedure);	↑ cortisol and ACTH at 30 and 60 min in CAST vs. NSAID, TA and SHAM groups.
Sutherland et al. [47]	36; 3 days	CAST; SHAM; TA (tetracaine); TA (L, B and adr). (TA administered post-incision, to spermatic cords and skin edge immediately prior to castration).	Trend (*p* = 0.06) ↑cortisol in CAST and TA piglets 0.5–1 h post castration but not at 90–180 min; ↑cortisol (*p* < 0.05) in TA (L,B and adr) piglets between 30–180 min post-castration.
Sutherland et al. [22]	70; 3 days	CAST; SHAM; SHAM with NSAID, SHAM with GA(CO_2_), CAST with NSAID, CAST with GA(CO_2_), CAST with both (NSAID = F, i.m. immediately prior to procedure)	Blood sampled before, and 30, 60, 120, and 180 min, 24 h, and 3 d after castration for cortisol, Substance P (0–180 min) and CRP (24 h–3 days). ↑ cortisol (30 min) in all CAST grps vs. SHAM grps. ↑cortisol (60–120 min) in CAST and CAST with NSAID vs SHAM grp. ↑CRP in CAST(trend) and CAST with GA (CO_2_) piglets. (↓CRP CAST with GA (CO2) vs. CAST piglets). ↑ SP in all piglet groups receiving GA (CO_2_).
Walker et al. [48]	85; 2–12	CAST; CAST with GA (Isofluorane)	↑ cortisol, ACTH and β-endorphins in CAST animals; NSD between anaesthetised and non-anesthetised groups despite obvious behavioural differences.
Kohler et al. [49]	21–28 days	CAST, CAST + GA (CO_2_/O_2_), CAST + GA(Halothane)	↑ cortisol, ACTH, β-endorphin; NSD between groups despite obvious behavioural differences.

**Table 2:** Summary of studies measuring motor response movements during castration.

Authors	Piglets N, Age	Castration Experimental Groups	Method	Significant Findings (*p* < 0.05)
Marchant-Forde et al. [50]	32; 2–8 days	Castration without anaesthesia (CAST); Cutting or tearing spermatic cord; sham-handled animals (SHAM)	Number of escape attempts (sequential kicks) during procedure	↑ escape attempts CAST vs. sham groups; no significant difference (NSD) in response between castration method (cut versus tear)
Horn et al. [59]	36; 10–14 days	CAST, local anaesthesia (LA) (LA = Lignocaine (L) administered i.t. +/− intrafunicularly prior to castration)	Relative proportion of resistance movements	↑ resistance movements in CAST, particularly prominent during spermatic cord cutting. ↓ in L-treated group
Leidig et al. [60]	61; 3–4 days	CAST; SHAM; LA; (LA = L or Procaine (P) i.t. prior to castration)	Ordinal scale measuring duration and intensity	↑ scores in CAST animals; ↓ scores in SHAM, L and P-treated animals
Saller et al. [55]	54; 3–7 days	(All - GA with minimum alveolar concentration isoflurane): CAST +/- saline or LA (L, P, bupivacaine or mepivacaine); SHAM	Ordinal scale measuring defensive limb movements	↑ scores in CAST+saline animals; ↓ scores in SHAM and all LA treated animals
Sheil et al. [32]	40; 3–7 days	CAST; topical wound anesthetic (TA), applied by wound instillation 30 s prior to excising testes.	Numerical rating scale	↑ scores in CAST piglets with traction on each teste and cutting of each spermatic cord; significantly reduced in TA-treated group
Walker et al. [48]	85; 2–12	CAST; CAST under general anaesthesia (GA) (Isofluorane)	Numerical rating scale	↑ scores in CAST piglets with skin incision and testis excision; significantly reduced in GA group
Keita et al. [28]	90; mean 5 days	CAST; SHAM; NSAID (NSAID = Meloxicam (M) i.m. 10–30 min prior to castration).	“Global” behaviour score (GBS) calculated from presence or absence of: foreleg; or hind leg; or other body movements; urine or faeces emission; tremors.	GBS was similar in the meloxicam and placebo groups. There was a behavioural response (i.e., global score of 1 or more) in more than 95% of all piglets in the study during castration
Hansson et al. [29]	564; 1–7 days	CAST; LA (L + adrenalin); NSAID(M); LA + M (Administration L and adr -i.t. 3–30 min prior, M-i.m. post castration)	Visual analogue scale	↑ scores in CAST animals; ↓ scores in L- and LM-treated animals

**Table 3:** Summary of studies measuring piglet vocal responses during castration.

Authors	Piglets Age, Number	Castration Experimental Groups	Measurement Method	Significant Findings (*p* < 0.05)
Wemelsfelder and van Putten [61]	4 weeks	Castration without anaesthesia (CAST); female litter mates	Calls highest in amplitude	Incising the scrotum did not result in a change in vocalisation, however pulling and cutting spermatic resulted in a marked ↑ in vocalisation
Puppe et al. [64]	19; 14 days	CAST	Rate of stress calls; STREMODO automated call monitoring system	↑ Stress calls (>1000 Hz) during surgical parts of castration procedure
Weary et al. [62]	102; 8–12 days	CAST; sham-castrated (SHAM)	Mean high (>1000 Hz) and low (<1000 Hz) calls	Significantly > high frequency calls in castrated vs. sham-handled piglets Greatest differences occurred during the severing of the spermatic cords and lesser differences when the scrotum was incised and the testicles extruded
Taylor and Weary. [68]	139; 7–10 days	CAST; SHAM	Mean high (>1000 Hz) and low (<1000 Hz) calls	Significantly > high frequency calls in castrated vs. sham-castrated piglets; pulling and severing produced highest call rate
Taylor et al. [63]	84; 3, 10, 17 days	CAST; SHAM	Mean high (>1000 Hz) and low (<1000 Hz) calls	Significantly > high frequency calls in castrated vs. sham-castrated piglets; No significant age effect observed on frequency of calls
Marchant-Forde et al. [50]	32; 2–8 days	CAST; (cutting or tearing spermatic cord); SHAM	Duration, mean frequency, and frequency of peak amplitude	Significantly > peak frequency of call in castrated piglets vs. sham-handled controls
White et al. [66]	172; 1–28 days	CAST; injectable lignocaine (L)	Frequency with highest decibel level (HEF)	Ligating cord produced ↑ HEF during castration; Significantly ↓ HEF in pigs treated with L
Marx et al. [67]	70; 7, 13, 19 days	CAST; L	12 variables	Calls classified into three types (screams, grunts squeals); 2 × number of screams in untreated castrates vs. treated
Leidig et al. [60]	61; 3–4 days	CAST; SHAM; L; Procaine (P)	STREMODO	CAST pain vocalisations significantly different from other treatment groups; no significant difference (NSD) between other groups
Kluivers-Poodt et al. [25]	160; 3–5 days	CAST; L; Meloxicam (M); L + M; SHAM	Temporal, waveform and spectral parameters	CAST piglets squealed longer and louder than piglets treated with L ± M; M-treated piglets similar to CAST
Keita et al. [28]	150; mean 5 days	CAST; M	Occurrence of vocalisation during castration recorded as ‘cry’, ‘growl’, or ‘silence’.	Vocalisation (crying) during castration occurred in 149 of the 150 piglets in the study. NSE of M treatment
Hansson et al. [29]	564; 1–7 days	CAST; L; M; L and M	Calls highest in amplitude	L and (L and M) piglets produced calls with significantly lower intensity than CAST, and M-treated piglets
Sutherland et al. [47]	36; 3 days	CAST; SHAM; topical anesthetic (TA); NSAID	STREMODO	Significant difference between SHAM piglets and castrated piglets (with or without treatment)
Sheil et al. [32]	40; 3–7 days	CAST; TA (with 30 s wait)	Peak dB and area under the dB/time (waveform) curve (AUC)	Significant reduction in vocal responses in TA (with 30 s wait) vs. CAST piglets during traction ad severance of first spermatic cord.
Sutherland et al. [22]	70; 3 days	CAST; SHAM; NSAID; GA (CO2); NSAID and GA (CO2)	STREMODO frequency of stress vocalisations	Percentage of stress vocalisations was greater (*p* < 0.05) in CAST and NSAID piglets than all other treatments.
Viscardi and Turner [65]	60; 5 days	CAST; SHAM; Buprenorphine (BUP); SHAM with BUP	Spectrograms from video recordings. Maximum; frequency (Hz), amplitude (μ), power (dB); and energy (dB) of each call was determined comparing skin marking, i.m. injection, skin incision, and castration	i.m. injection and castration (pulling and severing the spermatic cord) induced vocalisations of ↑ frequency (Hz), power (dB), and amplitude (u) and/or energy, than skin incision, and/or spray marking/sham-handling—all groups. NSE of Buprenorphine treatment.

**Table 4:** Summary of behavioral studies in neonatal piglets following castration.

Authors	Piglets Number, Age,	Castration Experimental Groups	Measurement Method	Significant Findings (NSE = no Significant Effect)
McGlone and Hellman [72]	20; 14 days	CAST; sham-handled (SHAM); Lignocaine (L)	Time lapse video recording; 3 h pre- and 3 h post-castration. Event recorder monitored general postures, position and feeding behaviour	3 h post-op ↓ standing; ↑ lying (away from heat); ↓ nursing in CAST piglets (low magnitude, no effect on weight gain)
McGlone et al. [66]	100; 1, 5, 10, 15 and 20 days	CAST; SHAM	Time lapse video recording. 24 h post-op. A digital timing and data summary program [106] was used to measure the duration of each behavior Ethogram based on [72]	↓ standing and ↑ lying and ↓ nursing 6 h post-castration in CAST piglets (low magnitude), no effect on weight gain.
Carroll et al. [58]	90, 3–12 days	CAST, SHAM	Time-lapse video recording (WJ-HD500A, 3-min scan sample immediately after castration for 2 h. Observed for “active” (running walking), lying, lying under the heat, sitting, sitting under the heat, standing, standing under the heat, and nursing (mutually exclusive).	NSE on the time that pigs spent nursing, lying, standing, or sitting, trend (*p* = 0.08) for CAST to be less active than SHAM. Overall age effect (*p* = 0.01) on the time that pigs spent standing, such that 3-day-old pigs stood more than 6-, 9-, or 12-day-old pigs. No effect on weight gain.
Taylor et al. [63]	84; 3, 10, 17 days	CAST; SHAM	Time-lapse video recording; Scan sampling. Proportion of total behaviours scored at 10 min intervalsMonitored general postures, location nursing and active/inactive behaviours.	↑ standing or sitting and ↓ lying 0–2 h post-castration in CAST piglets; ↓ lying and ↑ nursing in next 22 h. No significant effect (NSE) position (all effects low magnitude no effect on weight gain)
Hay et al. [51]	84; 5 days	CAST; SHAM (Previously tail-docked)	Detailed ethogram: posture, location, non-specific and pain-specific activity/behaviours and social isolation/desynchronization. Direct observation. Scan sampling every 10 min immediately post-op and 2 h each morning and evening for 5 days	First 2.5 h; ↑ “pain-specific” behaviours (prostration, huddled up, stiffness and trembling), ↑tail wagging ↑isolation and desynchronization ↓ suckling/udder massage, ↑ awake inactive in CAST piglets. 2–4 days; ↑scratching, tail wagging. Through-out; ↑walking and huddled up. Low magnitude ↑ kneeling otherwise NSE on postures or weight gain
Moya et al. [54] Exp 1	20; 5–8 h post-op	CAST; SHAM	Direct observation, Scan sampling every 3 min for 3 h (5–8 h post op); ethogram based on [51]	↑ total “pain-specific” behaviours (↑huddling up); ↓ walking; ↑ udder massage/exploratory activity and scratching (NSE posture or position)
Moya et al. [54] Exp 2	20; 4 days	CAST; SHAM	Direct observation, Scan sampling every 3 min for 2 h each morning and evening for 4 days; ethogram based on [51]	↑ total “pain-specific” behaviours (↑huddled up; tremors; spasms) first 0–2.5 h; Later time points ↓ sitting and ↑ trend for isolation. (Tail-wagging not recorded)
Keita et al. [28]	150; mean 5 days	CAST; Meloxicam (M);	Direct observation, Focal assessment (presence/absence) of “pain-specific” behaviours” based on [51] (prostration, tremors (trembling), tail movements and isolation) at 30 min, 1, 2, 4, and 24 h post-castration;	Greater proportion showed total global pain score ‘0′ in M vs. CAST at 2 and 4 h (NSE 30 min, 1 or 24 h)
Kluivers-Poodt et al. [27]	160; 3–5 days	CAST; SHAM; unhandled; Lignocaine (L); M; L and M. (Piglets not tail-docked)	Direct observation, Scan sampling; 12 min intervals for 3.5 h each morning and afternoon for 4.5 days; Ethogram based on [51], tail-wagging scored separately from other pain-specific behaviours	↑“pain-specific” behaviours (2–6 h), ↑ tail-wagging in L group (3 days). ↑ sleeping and inactive behaviours in all groups in first 2–6 h post-castration. NSE suckling behaviour
Hansson et al. [29]	398; 1–7 days	CAST; L; M; (L and M)	Direct observation scan sampling; each 10 min for 70 min. Ethogram based on [51,54,61].	(L and M) group; ↓ total “pain-specific” behaviours (huddled up, stiffness, prostration, tremors/trembling, spasms, scratching) day 1 post castration.
Gottardo et al. [45]	196; 4 days	CAST; SHAM; 2% topical tetracaine hydrochloride (THCL); 6% THCL; M; ketoprofen (K); tolfenamic acid	Direct observation, scan sampling 1 min intervals for 0–30 min and 60–90 min post-castration; Ethogram based on [54]	↑ total “pain-specific” behaviour (tremors, scratching, hunching, tail-wagging) CAST group, ↑isolation CAST and THCL groups; ↑ standing inactive all groups except K and SHAM in first 30 min. NSE 60–90 min period
Sutherland et al. [47]	36; 3 days	CAST; SHAM; topical wound anesthetic (L,B and adr administered to wound during procedure)	Direct observation, 1 min scan sampling for 180 min post-castration; ethogram based on [54,106,107] incl. Lying with or without contact, suckling behavior, general postures and “pain-specific” behaviours (huddled up or scratching).	↑ lying without contact in the CAST group
Sutherland et al. [22]	70; 3 days	CAST; SHAM; General anaesthesia (GA)-(CO_2_/O_2_); NSAID	1 min scan sampling 0–30, 60–90 and 120–150 min post castration; ethogram as per [47]	↑ lying without contact; CAST first 30 min thereafter CAS and CO2 piglets spent more time lying without contact than other treatments. ↑ total “pain-specific” behavior (scratching, huddling, hunched), CAS + CO2, 0–30 min.
Viscardi et al. [71]	19; 5 days	CAST; (M and EMLA^®^ cream), (M and Placebo cream), (saline and EMLA^®^), (saline and placebo), prior to surgical castration, tail docking and i.m. iron injection.	Video recording 1 h pre-; 0–8 h and 24 h post-castration; analysed 15 min per hour, ethogram based on [51], behaviours analyzed separately, and grouped into “active” and “inactive” categories	↑ inactive behaviors and ↑tail-wagging all groups first 6 h post castration and docking as compared with pre-castration and docking. ↑isolation in piglets castrated without treatment as compared with treatment groups. (NSE individual “pain-specific” behaviours other than tail wagging, however small sample size).
Viscardi and Turner [69]	120; 5 days	CAST; SHAM; M; K. (Piglets not tail-docked)	Video recording 1 h pre-; 0–8 h and 24 h post-castration; analysed 15 min per hour; ethogram adapted from [51] as above. Behaviours analyzed separately, and grouped into “active” “inactive” and “pain” categories. “Pain” included; trembling, stiffness, spasms, tail wagging, and rump scratching	All groups; At 0 h, ↑active behaviours; At 5 h,↑ suckling; At 7 h ↑sleep compared with pre-op. CAST, M, and K groups; At 2, 7 and 24 h post-castration ↑tail-wagging and “pain” behaviour,. (Note “pain” category included tail-wagging). (NSE scratching or other individual pain-specific behaviours)
Yun et al. [23]	143; 5 days	CAST; No castration (left in trolley) (NoC); M; L; GA (isoflurane and M)	Video recording, analysed 10 min/h, pre- (−1 h), 0, 1, 2 h and 24 and 36 h post-castration; ethogram based off [51,54,70] and others; behaviors analysed separately. (Scans were delayed if piglets were sleeping or feeding).	Comparing pre and post castration, behaviours different only during the first 10 min observation in both CAST and NoC piglets, but not different after 1 h. Comparing CAST versus NoC- at 0 h, ↑ prostration and ↓aggression and tail wagging in CAST. At 1 h ↑prostration and abnormal walking, otherwise NSE at any time points. M, L and GA piglets; 0–2 h ↑leg crossing vs. NoC, ↓abnormal walking and prostration M v CAST. At 2 h ↑ tail wagging GA vs. NoC. Otherwise NSE
Viscardi and Turner [65]	60; 5 days	CAST; SHAM (+saline); CAST and buprenorphine; SHAM and buprenorphine (Piglets not tail docked)	Video recording 1 h pre-; 0–8 h and 24 h post-castration; ethogram based on [51] behaviors analyzed separately, and grouped into “active” and “inactive” and “pain” categories. “Pain” included: trembling, stiffness, spasms, tail wagging, and rump scratching	All groups; ↑sleeping and ↓ walking, standing and active behaviours 4–7 h as compared with 0 h. ↑ active behaviours Buprenorphine versus other groups 0–7 h. ↑ tail-wagging and “pain” behaviours 24 h post-castration, CAST versus SHAM group. NB: “pain” category included tail-wagging.
Burkemper et al. [24]	235; 3–7 days	CAST; Lignocaine spray (LS); oral M; LS and oral M	direct observation, scan sampling each 5 min for 5 h period for 3 days post op; total pain and 5 “pain-specific” behaviours based on [51] (tail wagging, trembling, huddled up, prostrated, scratching)	↑ total pain-specific behaviours max 0–1 h post castration. No significant difference observed in behaviour between treatment groups. (Trend for ↑pain-specific behaviour in LS group)
Langhoff et al. [35]	245; 4–6 days	CAST; M, flunixin (F), metamizole or carprofen, respectively, administered 15 to 30 min prior.	Post-surgical behaviour (0–60 min and 180–240 min after castration/handling)	Tail wagging, drooping the tail, and changing the position were reduced in M and F piglets

**Table 6:** Summary of studies assessing wound sensitivity after castration in neonatal piglets. (Arrows indicate statistical significance (p<0.05). NSD = No significant difference of treatment).

Authors	Piglets Age, Number	Castration Experimental Groups	Measurement Method	Significant Findings
Lomax et al. [33]	40; 3–5 days	Castration without anaesthesia (CAST); sham-castrated (SHAM); topical wound anesthetic (TA)(TA = 5% Lignocaine (L), 0.5% Bupivacaine (B) and adrenalin 1:2000 (adr) administered via wound instillation)	von Frey filaments (4 g and 300 g) and 18 G needle; testing immediately after, 1 min, and every 30 min up to 4 h; grading on NRS for involuntary motor response	Vs SHAM: ↑ sensitivity scores post castration CAST, 30 min – 4h; L ↑ 1 – 4 h. (TA NSD). Vs CAST ↓ sensitivity post-castration; L 30 – 90 min, TA 30 min- 4h.
Gottardo et al. [45]	196; 4 days	CAST; SHAM; NSAID (NSAID = meloxicam or ketoprofen or tolfenamic acid); TA (TA = 2% topical tetracaine (THCL) hydrochloride or 6% THCL administered pre-and post-operatively.	Pressure algometry with pressure ranging between 0.1–20 kg/cm^2^; testing 300 min post-castration	Vs SHAM: ↑ sensitivity to pressure post castration a) immediately; CAST and TA, b) after 15 min TA; from 60–300mins all groups. Vs CAST ↓ sensitivity to pressure in NSAID -treated piglets immediately post castration, otherwise NSD.
Sheil et al. [32]	40; 3–7 days	CAST; TA (TA = L,B and adr, administered via wound instillation);	von Frey filament (300 g) and pin-prick; testing 1 min and 1, 2, 4, 8, 12, and 24 h post-castration; grading on NRS for involuntary nociceptive response based on [32]	Statistically significant difference between treated and CAST groups at 1 min and up to 2 h post-castration with ↓ sensitivity in TA vs CAST group.

**Table 5:** Summary of statistically significant results (*p* < 0.05) from behavioral studies examining posture, position, activity and pain-related behaviours in neonatal piglets post-castration as compared with sham-handled piglets. (Arrows indicate statistical significance. NSE = No significant effect of treatment).

Authors		Posture			Position		Activity		Pain-Specific Behaviours	Weight Gain
Compared to Sham-Castrated Piglets < 2 Weeks of Age	Time of Post-Operative Assessment	Lying	Standing	Sitting	Isolation	Heat-Lamp/(Position in Crate)	Suckling/Nursing	Active/Inactive Behaviours		
McGlone and Hellman [72]	0–3 h	Minor ↑	Minor ↓	-	-	Minor ↓	Minor ↓	-	-	NSE
McGlone et al. [70]	0–6 h	Minor ↑	Minor ↓	-	-	Minor ↓	Minor ↓	-	-	NSE
Taylor et al. [63]	0–2 h	Minor ↓	Minor ↑	Minor ↑	NSE	NSE	NSE	-	-	-
	2–22 h	Minor ↓	NSE	NSE	NSE	NSE	Minor ↑			
Carroll et al. [58]	0–2 h	NSE	NSE	-	-	NSE	NSE	NSE	-	NSE
Hay et al. [51]	5 days	NSE	NSE	NSE	↑ 0–2.5 h	NSE	↓ 0–2.5 h	↑ awake inactive 0–2.5 h ↑ walking through-out	↑ total, ↑prostration, stiffness, trembling tail-wagging 0–2.5 h; ↑ huddled up ↑ scratching tail-wagging for 2–4 days	NSE
Moya et al. [54] Exp 1	5–8 h	NSE	NSE	NSE	↓	NSE	↑ Trend	↓ walking and ↑ exploratory behaviour	↑ total, ↑ huddled up ↑scratching	NSE
Moya et al. [54] Exp 2	4 days	NSE	NSE	↓ (through-out)	↑ Trend	NSE	NSE	Trend ↓ active behaviours	↑ total, ↑ huddled up, spasms, trembling (0–2.5 h)	NSE
Keita et al. [28]		-	-	-	-	-	-	-	↑ total (prostration, tremors/trembling, tail movements and isolation) 2 and 4 h post castration.	NSE
Kluivers-Poodt et al. [27]		NSE	NSE	NSE	NSE	NSE	NSE	↑sleeping and inactive behaviours (all groups) Day 1 p.m.	↑ total Day 1 p.m. (2–6 h) (huddled, stiffness, spasms, prostrated, tremors/trembling) ↑tail wagging Day 5 a.m only (Lidocaine group ↑tail wagging day 1–3, and 5 a.m).	NSE
Gottardo et al. [45]		NSE	NSE	NSE	↑ 30 min post-op	NSE	NSE	↑ standing inactive (30 min post-op)	↑ total (tremors, hunching, scratching, tail-wagging) for 30 min post-castration	NSE
Sutherland et al. [47]		-	-	-	↑ 180 min post-op	-	NSE	NSE	NSE (limited range = huddled up or scratching)	NSE
Sutherland et al. [22]		-	NSE	NSE	↑ 30 min post-op	-	NSE	NSE	NSE (limited range = huddled up or scratching)	NSE
Viscardi et al. [71]	Day-1–24 h	NSE	NSE	NSE	↑0–7 h (versus Day 1)	-	NSE	↑ 0–7 h (versus Day-1)	↑ tail-wagging otherwise NSE (individual)	-
Viscardi and Turner [65]	Day-1–24 h	NSE (various time effects)	NSE (various time effects)	NSE	NSE	-	NSE	↑sleeping and lying ↓walking, standing and active behaviours 4–7 h as compared with 0 h all groups.	↑ total 24 h (↑tail-wagging)	NSE
Viscardi and Turner [69]	Day-1–24 h	Various time effects	Various time effects	NSE	↑ various	-	NSE	↑active behaviours 0 and 24 h as compared with various other times both groups	↑ total and tail-wagging 2, 7 and 24 h (Note total “pain” score predominantly increased due to increased tail-wagging)	-
